# Supplementary material for: Altered Genome-Wide DNA Methylation in Peripheral Blood of South African Women with Gestational Diabetes Mellitus
Source: Int J Mol Sci. 2019 Nov 20;20(23):5828. doi: 10.3390/ijms20235828 (PMC6928622; doi:10.3390/ijms20235828)
Supplement: Supplementary file 1 [file ijms-20-05828-s001.zip › Supplementary figures/Figure S1.docx]

**
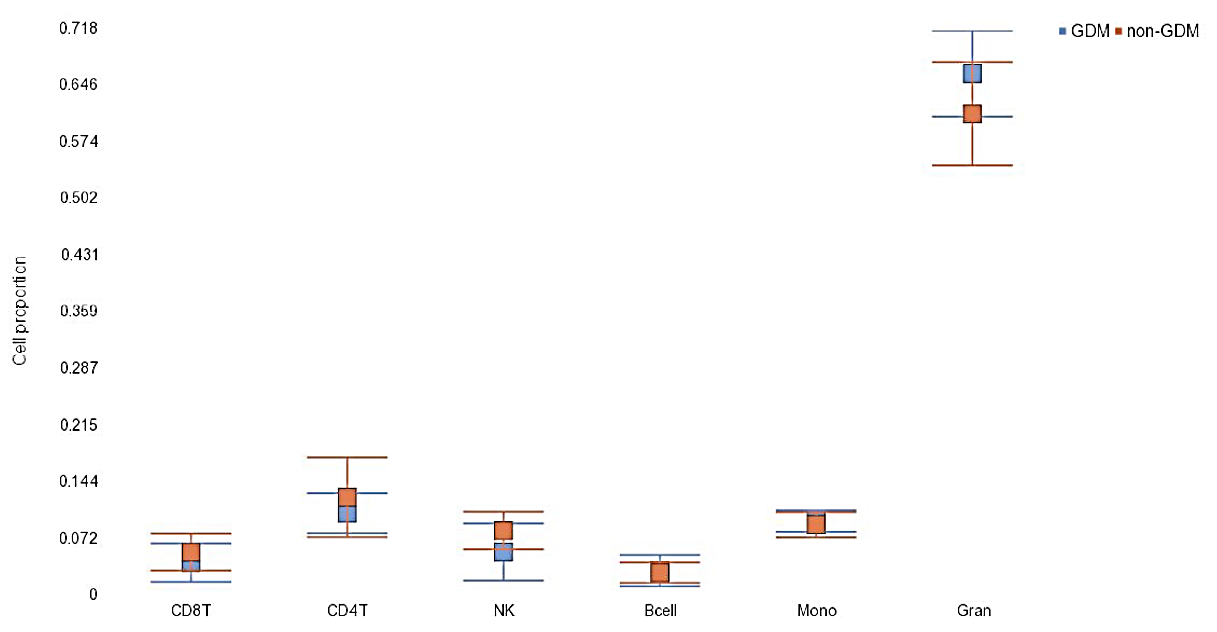
**

**Supplementary figure S1.** Comparison of six major peripheral blood cell components in GDM and non-GDM women. No significant difference was observed between cells types. All Data points are presented as means with standard deviation. *p*<0.01 is considered significant.
